# Clinical Characteristics and Risk Factors of Immune Checkpoint Inhibitor‐Related Myocarditis: A Real World Observational Study

**DOI:** 10.1002/clc.70312

**Published:** 2026-04-20

**Authors:** Mei Zhan, Qinran Long, Ran Liu, Litao Huang, Bin Wu, Ting Xu, Jinhan He

**Affiliations:** ^1^ Department of Pharmacy, West China Hospital Sichuan University Chengdu Sichuan China; ^2^ West China School of Pharmacy Sichuan University Chengdu Sichuan China; ^3^ Department of Pharmacy, Institute of Metabolic Diseases and Pharmacotherapy, West China Hospital Sichuan University Chengdu Sichuan China; ^4^ Engineering Research Center of Medical Information Technology, Ministry of Education West China Hospital of Sichuan University Chengdu Sichuan China; ^5^ Department of Clinical Research Management, West China Hospital Sichuan University Chengdu Sichuan China

**Keywords:** cardiotoxicity, immune checkpoint inhibitors, immune‐related adverse events, myocarditis

## Abstract

**Background:**

Rare occurrence, immune checkpoint inhibitors (ICI)‐related myocarditis are poorly documented in the literature.

**Research Design and Methods:**

We conducted a retrospective review of patients who had ICI until June 2023. Patient follow‐up was extended until death or on May 2024. The primary outcome was the incidence of suspected ICI‐related myocarditis. Logistic regression was used to investigate the associations between clinical characteristics and suspected ICI‐related myocarditis.

**Results:**

Among the included 8199 patients, 1638 (19.98%) patients developed suspected ICI‐related myocarditis. Logistic regression revealed that thymomas and thymic carcinomas (OR 2.242; [95% CI, 1.132–4.440], *p* = 0.021), lung cancer (OR 1.259; [95% CI, 1.119–1.416], *p *< 0.001), older patients (OR 1.021; [95% CI, 1.016–1.026], *p *< 0.001), male (OR 1.213; [95% CI, 1.061–1.388], *p* = 0.005), block two or more immune checkpoints (OR = 1.391 [95% CI 1.058–1.828], *p* = 0.018), combined with hypertension (OR = 1.326 [95% CI 1.162–1.513], *p* < 0.001), or hyperlipidemia (OR = 1.656 [95% CI 1.302–2.107], *p* < 0.001) were associated with higher risk of suspected ICI‐related myocarditis.

**Conclusions:**

This large, real‐world cohort demonstrates that the incidence of suspected ICI‐related myocarditis may be underestimated in previous literature. Routine cardiac surveillance is needed in high‐risk patients receiving ICI therapy.

**Trial Registration:**

Registered at https://www.chictr.org.cn (identifier: ChiCTR2300075974).

## Graphical abstract

This real‐world study reveals a 19.98% incidence of suspected immune checkpoint inhibitor‐related myocarditis, significantly higher than previously reported. Key risk factors include thymic malignancies, lung cancer, older age, male, block two or more immune checkpoints, and preexisting hypertension or hyperlipidemia, underscoring the need for routine cardiac surveillance in high‐risk patients.

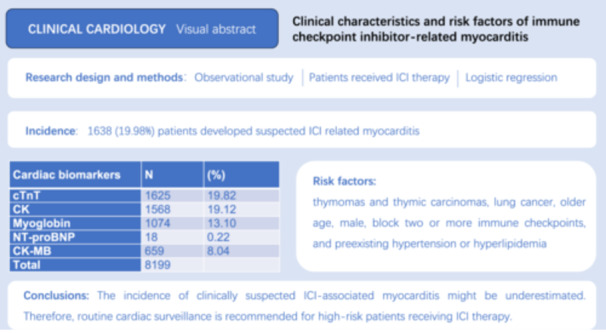

## 1 Introduction

Immune checkpoint inhibitors (ICI) work indirectly via the host's adaptive and innate immune system by blocking T cell checkpoints to recognize and eradicate tumor cells [1]. The primary targets for checkpoint inhibition include programmed cell death protein 1 (PD‐1), programmed cell death ligand 1 (PD‐L1), and cytotoxic T‐lymphocyte antigen 4 (CTLA‐4). ICI have dramatically improved clinical outcomes for multiple tumor types, including melanoma, non‐small cell lung cancer, renal cell carcinoma, and urothelial carcinoma [2]. ICI have revolutionized cancer treatment but are associated with a unique spectrum of organ‐specific inflammatory toxicities that can affect multiple organ systems. Timely diagnosis and appropriate management are vital to optimize the quality of life and long‐term outcomes of patients receiving ICI. Aggregating clinical data, real‐world data (such as data on pharmacovigilance or from electronic health records), and multi‐omics data are crucial for a comprehensive understanding of their clinical outcomes and mechanisms [3]. Clinical trials and real‐world data primarily focus on common immune‐related adverse events (irAEs). So common toxicities are well described in the literature, and their management protocols have been established [4, 5]. But due to the limited evidence, rare irAEs are less understood and pose diagnostic and therapeutic dilemmas.

ICI‐related myocarditis is a rare but potentially life‐threatening irAE. The incidence of ICI‐related myocarditis varies between 0.04% and 2.4%, with a potential mortality rate of up to 50% [6, 7, 8, 9, 10]. The incidence of ICI‐related myocarditis may be underestimated due to several factors, such as nonspecific symptoms, the lack of specific markers, potential confusion with other heart conditions, and inconsistent diagnostic criteria recommended in the guidelines [4, 11, 12, 13]. The clinical presentation of ICI‐related myocarditis may range from asymptomatic cardiac biomarkers elevation to life‐threatening cardiac compromise [14]. Current data about prevalence of ICI‐related myocarditis are limited, further research is required to define the incidence, characteristics, and risk factors of ICI‐related myocarditis. Therefore, to better define the incidence, characteristics, and risk factors of ICI‐related myocarditis, we conducted a real‐world observational study. Herein, “real‐world data” are derived from routine clinical practice, reflecting heterogeneous patient populations and unstructured, non‐protocol‐driven treatments, thereby complementing findings from controlled clinical trials.

## 2 Methods

### 2.1 Study Design and Patient Selection

This single‐center prospective observational study of patients who received at least one administration of ICI at West China Hospital, Sichuan University, from 2014 to June 2023. We extracted basic information of patients who might receive ICI from a Big Data platform at the West China Hospital, Sichuan University, based on inclusion criteria. Medical records were manually reviewed to confirm the inclusion criteria and extract information that could not be directly obtained from big data platforms. Patients were assessed at death or on May 7, 2024, whichever occurred first. This study was approved by the Biomedical Ethics Review Committee of the West China Hospital, Sichuan University (2023 Review [No. 1064]).

### 2.2 Definitions and Outcomes

American Society of Clinical Oncology (ASCO) guidelines defined grade 1 ICI‐related myocarditis as abnormal cardiacbiomarkers or electrocardiogram (ECG) without symptoms [9, 12]. Society for Immunotherapy of Cancer (SITC) clinical practice guideline suggests that patients developed new cardiac symptoms, new cardiac arrhythmias, new heart blocks, or cardiac lab findings (e.g., asymptomatic troponin elevation) within 12 weeks after receiving an ICI therapy should be diagnosed as ICI‐induced myocarditis [13]. According to the recently updated clinical practice guidelines, the criteria for suspected ICI‐related myocarditis can either be newly diagnosed myocarditis or a clinical diagnosis established by newly abnormal values of troponin after ICI therapy. We excluded myocarditis with a clear etiology, where the cause was not ICI.

The primary endpoint was the incidence of suspected ICI‐related myocarditis, and secondary endpoints included the incidence of abnormal myocardial markers after ICI therapy and the risk factors for ICI‐related myocarditis. Patients who developed new myocardial markers abnormalities after ICI treatment, excluding baseline abnormalities. The myocardial markers, included cardiac troponin T (cTnT), N‐terminal pro‐B‐type natriuretic peptide (NT‐proBNP), myoglobin, creatine kinase (CK), and creatine kinase‐myocardial band (CK‐MB).

### 2.3 Data Analysis and Statistics

Data on demographic characteristics, cancer types and laboratory test findings and so on were obtained from the Big Data platform at West China Hospital, Sichuan University. Details of ICI therapy were collected from the electronic medical records. Myocardial markers levels were measured in the clinical laboratories at West China Hospital, Sichuan University.

Statistical analysis was carried out using SPSS version 29.0 software. Continuous variables that were not normally distributed were expressed as medians and interquartile ranges (IQR), and continuous variables with normally distribution were described as means with standard deviations (SD). Categorical variables are presented as numbers (percentages). Univariate and multivariate logistic regression were performed to determine those variables that significantly contributed to suspected ICI‐related myocarditis. Predictor variables for multivariate analysis were prespecified based on clinical experience or significance in univariable regression. A two‐tailed Student's *t*‐test was used to determine differences between groups. Univariate analysis identified variables with *p*‐value < 0.20, which were then included in multivariate regression analysis. The variance inflation factor (VIF) was used to detect multicollinearity. Results of the multivariate logistic analysis were described as adjusted odds ratio (OR) and its 95% confidence intervals (95% CI), and *p*‐value < 0.05 were considered statistically significant.

To evaluate the robustness of our primary findings, we conducted sensitivity analyses through multiple approaches. First, we compared results from the full multivariable logistic regression with those from stepwise selection models to assess effect estimate consistency. Second, we repeated analyses after excluding participants with high missing data rates or extreme values to evaluate the impact of these exclusions on the results.

## 3 Results

### 3.1 Incidence of Suspected ICI‐Related Myocarditis

**FIGURE 1:**
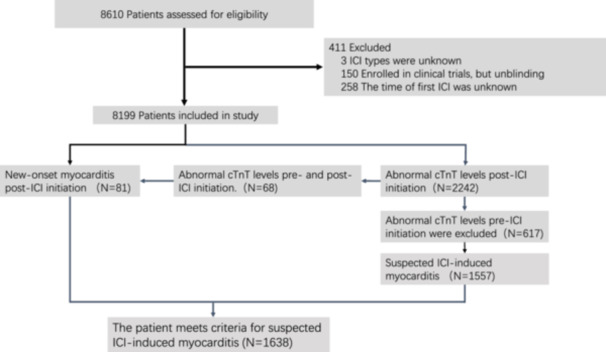
Flow chart of patients receiving immune checkpoint inhibitors. Screening, Enrollment, and Results. cTnT, cardiac troponin T; ICI, immune checkpoint inhibitors.

The initial study population included 8610 patients who received ICI between October 2014 and June 2023. We excluded 411 patients who lack key information, such as the time of first ICI use. Finally, there were 8199 patients at the time of this retrospective review. The study population (*n* = 8199) was predominantly male (74.08%) and received ICI for lung cancer (37.97%), with a median age of 59.20 years [IQR 52.0–67.70 years]. In total, 1638 (19.98%) patients met the diagnosis criteria for suspected ICI‐related myocarditis, of which only 81 (0.99%) patients were diagnosed with myocarditis after ICI treatment, while 1557 (18.99%) patients without diagnosis of myocarditis experienced newly abnormal values of cTnT after ICI therapy. The research process is detailed in Figure 1. A total of 2242 patients experienced abnormal cTnT levels after medication, of which 617 patients had abnormal levels of troponin before medication. Sixty‐eight patients had abnormal cTnT levels before and after ICI therapy, and were classified as suspected ICI‐related myocarditis due to newly diagnosed myocarditis after ICI therapy. One patient with normal cTnT levels (< 21.00 ng/mL) was diagnosed with suspected ICI‐related myocarditis due to sinus tachycardia shown on the electrocardiogram, NT‐proBNP elevation, and decreased activity tolerance. The clinical characteristics of the patients are summarized in Table 1.

**TABLE 1:** Baseline patient demographics and clinical characteristics.

Characteristic	All	Non‐suspected ICI induced myocarditis	Suspected ICI induced myocarditis
Total, *N*	8199	6561	1638
Age, years, median [IQR]	59.2 [52.0, 67.7]	58.3 [50.5, 66.6]	62.6 [55.2, 69.3]
Sex			
Female *n* (%)	2125 (25.92)	1777 (27.08)	348 (21.25)
Male *n* (%)	6074 (74.08)	4784 (72.92)	1290 (78.75)
Tobacco use	3296 (40.20)	2591 (39.49)	705 (43.04)
Alcohol use	2399 (29.26)	1892 (28.84)	507 (30.95)
Hypertension *n* (%)	1696 (20.69)	1244 (18.96)	452 (27.59)
Hyperlipidemia *n* (%)	343 (4.18)	238 (3.63)	105 (6.41)
The type of ICI			
PD‐1	7264 (88.60)	5849 (89.15)	1415 (86.39)
PD‐L1	642 (7.83)	494 (7.53)	148 (9.04)
CTLA‐4	1 (0.01)	1 (0.02)	0 (0.00)
≥ 2	292 (3.56)	217 (3.31)	75 (4.58)
Primary cancer			
Other	4913 (59.92)	4059 (61.87)	854 (52.14)
Thymus	41 (0.50)	29 (0.44)	12 (0.73)
Lung	3112 (37.96)	2371 (36.14)	741 (45.24)
Multiple primary tumors	133 (1.62)	102 (1.55)	31 (1.89)

Abbreviations: CTLA‐4, cytotoxic T‐lymphocyte antigen 4; ICI, immune checkpoint inhibitors; IQR, interquartile ranges; PD‐1, programmed cell death protein 1; PD‐L1, programmed cell death ligand 1.

### 3.2 The Incidence of Abnormal Myocardial Markers

Of the 8199 patients, only 26 patients did not undergo myocardial markers measurements during the study period. A total of 2913 (35.53%) patients had records of myocardial markers exceeding the reference value during the study period. The most frequently monitored myocardial marker during medication is CK, with 149 643 tests performed. The most common abnormality in myocardial markers was cTnT elevation, with a total of 1625 (19.82%) patients experiencing elevated cTnT levels. During the study period, 49 035 cTnT tests and 48 105 myoglobin tests were conducted, respectively. A total of 1074 (13.10%) patients experienced abnormal myoglobin levels. The incidence of abnormal myocardial markers was summarized in Table 2.

**TABLE 2:** Incidence of abnormal myocardial markers.

Cardiac biomarkers	*N*	(%)
cTnT	1625	19.82
CK	1568	19.12
Myoglobin	1074	13.10
NT‐proBNP	18	0.22
CK‐MB	659	8.04
Total	8199	

Abbreviations: CK, creatine kinase; CK‐MB, creatine kinase‐myocardial band; cTnT, cardiac troponin T; NT‐proBNP, N‐terminal pro‐B‐type natriuretic peptide.

ECG results were further analyzed in patients with elevated cTnT levels. Among the 1625 patients, 1171 underwent ECG testing before ICI therapy, and 1055 received ECG monitoring after ICI therapy. Among these, 829 patients had abnormal ECG findings after treatment. Specifically, 283 of them had abnormal ECG results before treatment, 323 had normal ECG results before treatment, and 223 had no prior ECG records.

### 3.3 Factors Associated With Suspected ICI‐Related Myocarditis

Table 3 shows the results of the logistic regression analysis that investigated risk factors for suspected ICI‐related myocarditis. Based on the results of the univariate logistic regression analysis, age, sex, combined with hypertension, hyperlipidemia or diabetes, primary cancer, and immune checkpoint targets should be included in multivariate logistic regression analysis (*p* < 0.2). No significant multicollinearity was detected among the predictors. All VIF values ranged from 1.009 to 1.343, significantly below the conventional threshold of 5. Collinearity diagnosis revealed no serious multi‐collinearity requiring correction among the variables in the model. In the multivariate analysis, the risk of suspected ICI‐related myocarditis was higher for thymomas and thymic carcinomas (OR 2.242; [95% CI, 1.132–4.440], *p* = 0.021), lung cancer (OR 1.259; [95% CI, 1.119–1.416], *p *< 0.001), older patients (OR 1.021; [95% CI, 1.016–1.026], *p *< 0.001), male (OR 1.213; [95% CI, 1.061–1.388], *p* = 0.005), block two or more immune checkpoints (OR = 1.391 [95% CI 1.058–1.828], *p* = 0.018), combined with hypertension (OR = 1.326 [95% CI 1.162–1.513], *p* < 0.001), or hyperlipidemia (OR = 1.656 [95% CI 1.302–2.107], *p* < 0.001).

**TABLE 3:** Logistic regression analysis of risk factors for suspected ICI‐induced myocarditis.

Characteristic	Univariable logistic regression				Multivariable logistic regression			
	*p*	OR	95% CI		*p*	OR	95% CI	
			Lower limit	Upper limit			Lower limit	Upper limit
Age	< 0.001	1.026	1.022	1.031	< 0.001	1.021	1.016	1.026
Sex	< 0.001	1.377	1.209	1.569	0.005	1.213	1.061	1.388
Hypertension	< 0.001	1.629	1.438	1.845	< 0.001	1.326	1.162	1.513
Hyperlipidemia	< 0.001	1.820	1.437	2.305	< 0.001	1.656	1.302	2.107
Diabetes	< 0.001	1.421	1.214	1.664	0.113	1.142	0.969	1.346
The type of ICI								
PD‐1	0.013				0.131			
PD‐L1	0.030	1.238	1.021	1.501	0.988	0.999	0.817	1.220
CTLA‐4	1.000	0.000	0.000		1.000	0.000	0.000	
≥ 2	0.009	1.429	1.092	1.869	0.018	1.391	1.058	1.828
Primary cancer								
Other	< 0.001				< 0.001			
Thymus	0.050	1.967	1.000	3.870	0.021	2.242	1.132	4.440
Lung	< 0.001	1.485	1.330	1.659	< 0.001	1.259	1.119	1.416
Multiple primary tumors	0.078	1.445	0.960	2.174	0.351	1.218	0.805	1.842

Abbreviations: CI, confidence interval; CTLA‐4, cytotoxic T‐lymphocyte antigen 4; ICI, immune checkpoint inhibitors; OR, odds ratio; PD‐1, programmed cell death protein 1; PD‐L1, programmed cell death ligand 1.

### 3.4 Factors Associated With Suspected ICI‐Related Myocarditis

Sensitivity analyses confirmed the robustness of the primary findings (Table 4). The key risk factors identified in the primary multivariable analysis—increased age, male, a history of hypertension or hyperlipidemia, and specific cancer types, including thymoma/thymic carcinoma and lung cancer—maintained statistical significance and consistent effect directions across all alternative model specifications. This includes both the stepwise selection model and the analysis excluding patients at age extremes. The association between treatment with two or more immune checkpoints and elevated risk remained significant in both the primary model and the analysis excluding patients at age extremes. However, this variable was excluded during the stepwise selection process due to a lack of statistical significance. Similarly, the variable “diabetes” was also excluded from the stepwise model and did not show a significant association in the other two models.

**TABLE 4:** Sensitivity analysis results.

Characteristic	Primary (full) model				Sensitivity analysis 1: stepwise selection				Sensitivity analysis 2: excluding age extremes (< 18 or > 85 years)			
	*p*	OR	95% CI		*p*	OR	95% CI		*p*	OR	95% CI	
			Lower limit	Upper limit			Lower limit	Upper limit			Lower limit	Upper limit
Age	< 0.001	1.021	1.016	1.026	< 0.001	1.021	1.016	1.026	< 0.001	1.021	1.016	1.027
Sex	0.005	1.213	1.061	1.388	0.004	1.22	1.067	1.394	0.006	1.207	1.055	1.38
Hypertension	< 0.001	1.326	1.162	1.513	< 0.001	1.354	1.189	1.541	< 0.001	1.332	1.167	1.521
Hyperlipidemia	< 0.001	1.656	1.302	2.107	< 0.001	1.671	1.314	2.125	< 0.001	1.682	1.322	2.141
Diabetes	0.113	1.142	0.969	1.346					0.12	1.14	0.967	1.343
The type of ICI												
PD‐1	0.131								0.139			
PD‐L1	0.988	0.999	0.817	1.22					0.983	0.998	0.816	1.22
CTLA‐4	1	0	0						1	0	0	
≥ 2	0.018	1.391	1.058	1.828					0.02	1.385	1.054	1.82
Primary cancer												
Other	< 0.001				< 0.001				< 0.001			
Thymus	0.021	2.242	1.132	4.44	0.022	2.218	1.121	4.392	0.021	2.236	1.129	4.428
Lung	< 0.001	1.259	1.119	1.416	< 0.001	1.266	1.129	1.42	< 0.001	1.256	1.117	1.413
Multiple primary tumors	0.351	1.218	0.805	1.842	0.376	1.205	0.797	1.824	0.415	1.19	0.783	1.811

Abbreviations: CI, confidence interval; CTLA‐4, cytotoxic T‐lymphocyte antigen 4; ICI, immune checkpoint inhibitors; OR, odds ratio; PD‐1, programmed cell death protein 1; PD‐L1, programmed cell death ligand 1.

## 4 Discussion

ICI‐related myocarditis are rare irAEs with unclear clinical features. Early clinical trials with CTLA‐4 and PD‐1 blockers rarely reported ICI‐related myocarditis, but the incidence of ICI‐related myocarditis is increasing in the real world [15, 16, 17]. Our study showed that the incidence of suspected ICI‐related myocarditis was significantly higher than that reported in literatures, with the diagnostic criteria from the ASCO. The main reason is the lack of clear diagnostic criteria for immune related myocarditis. ICI‐related myocarditis poses a diagnostic challenge due to the limitations of current diagnostic tools and overlapping clinical presentations with cardiovascular disease and cancer‐related complications [9, 18]. Mirabel 2024 revealed that early major cardiovascular occurred events in 18.4% of ICI‐treated patients (mean onset: 40.1 days), demonstrating that systematic monitoring uncovers higher cardiotoxicity rates than historically reported, particularly for heart failure and arrhythmias [19]. Furukawa 2023 identified elevated troponin I levels in 14.3% of participants (*n* = 18/126), of which 10.3% (*n* = 13) developed clinically suspected myocarditis based on a guideline‐recommended scoring system for myocarditis [20, 21]. Van den Berg 2024 showed that 26 patients (16%) exhibited troponin elevations during ICI therapy, but only eight patients (5%) developed ICI‐myocarditis [22]. These studies demonstrated that ICI‐associated myocardial injury occurs more frequently than previously recognized, but can often be managed without treatment interruption when detected early through vigilant monitoring. Current guidelines on management of immunotherapy‐related toxicities exhibit significant heterogeneity in diagnostic criteria for ICI‐related myocarditis. According to the guideline on toxicities from immunotherapy of European Society for Medical Oncology (ESMO), the diagnosis of ICI‐related myocarditis depends on a combination of clinical, electrocardiographic, cardiac biomarker, and imaging, such as echocardiogram and cardiac MRI (CMR) and endomyocardial biopsy [4]. Additionally, endomyocardial biopsy is the criterion standard diagnostic test, but is invasive and not routinely performed due to its risks and sampling variability [12, 23]. Although cTnT play a crucial role in diagnosing cardiovascular complications during ICI treatment, our study showed that cTnT elevations were often asymptomatic, and did not always lead to myocarditis diagnosis. Notably, our results demonstrated that a considerable proportion of patients with elevated cTnT also presented with ECG abnormalities, including cases where these abnormalities developed de novo after ICI therapy. This pattern of concurrent biomarker elevation and ECG changes, even in the absence of overt symptoms, is suggestive of subclinical myocardial injury and represents a critical, potentially actionable phase in the evolution of ICI‐related cardiotoxicity. This observation reinforces the imperative for combined monitoring strategies during ICI therapy. ASCO guidelines recommend asymptomatic troponin elevation was classified as grade 1 severity myocarditis [12]. However, troponin may no longer be cardiac‐specific and many other factors may lead to its elevation in addition to myocarditis [24]. Therefore, our study may overestimate the incidence of suspected ICI‐related myocarditis. Among 1625 patients with troponin elevation after ICI treatment, 11 patients were diagnosed with myocardial infarct, five patients were diagnosed with myocardial injury, one patient was diagnosed with myocardial ischemia, one patient was diagnosed with myocardial fat infiltration, and six patients were diagnosed with two diseases simultaneously: cardiomyopathy, myocarditis, myocardial infarction, and myocardial ischemia. Most patients experience unexplained elevation of cTnT, which also indicates that the spectrum of ICI‐related myocarditis remains understudied and a significant amount of mild myocarditis may be overlooked. We applied ASCO criteria despite pre‐existing abnormalities, this approach may overestimate suspected ICI‐myocarditis incidence but reflects real‐world decision‐making where endomyocardial biopsy availability is limited.

Our study has some limitations. First, this is a retrospective, single‐center study and the study population may not be representative of the broader population. In addition, because retrospective study relies on historical data, which may be incomplete, reducing the reliability of the findings. Despite these limitations, retrospective study remains a useful tool to evaluate drug safety [25]. ICI‐related myocarditis are rare ir‐AEs, and the available data are mostly limited to case reports and series, pharmacovigilance studies and meta‐analyses. We included information on patients who have received ICI in a large tertiary hospital over the past 10 years in our study. To our knowledge, this is the largest retrospective cohort study to analysis of the incidence of ICI‐related myocarditis diagnosed according to guidelines on ICI‐related adverse events. Therefore, it still can provide important information on the epidemiology and possible predictive factors of ICI‐related myocarditis. Second, elevation of cTnT can be attributed to many factors, but using clinical diagnosis to identify ICI‐related myocarditis may underestimate incidence if events are underdiagnosed or diagnosis incorrectly. If only patients were newly diagnosis of myocarditis after ICI treatment were considered to have suspected ICI‐related myocarditis, the incidence of ICI‐related myocarditis was 1.60% (131/8199). Although several aspects of ICI‐related myocarditis remain unclear, we have developed diagnostic criteria based on the ICI management guidelines to identify ICI myocarditis as sensitively as possible. Third, owing to the lack of appropriate negative control exposures in our dataset, negative control analysis was not feasible in our study. This limitation limits our ability to assess potential biases, such as surveillance bias (due to differential cardiac monitoring) or residual confounding (from unmeasured clinical factors). We acknowledge the possibility of residual confounding, particularly from unmeasured treatment‐selection factors or concomitant therapies, and this should be considered when interpreting the results.

## 5 Conclusions

The incidence of ICI‐related myocarditis in clinical practices may be significantly higher than that reported in previous literature and therefore may be greatly underestimated. Routine cardiac surveillance is needed in high cardiovascular risk patients receiving ICI therapy.

## Author Contributions

Conceived and designed the analysis: Mei Zhan, Qinran Long, and Jinhan He. Collected the data: Mei Zhan, Qinran Long, and Ran Liu. Contributed data or analysis tools: Litao Huang, Bin Wu, and Ting Xu. Performed the analysis: Mei Zhan and Qinran Long. Wrote the paper: Mei Zhan, Qinran Long, and Jinhan He.

## Ethics Statement

This retrospective study was conducted following the basic principles of the Declaration of Helsinki and has been approved by the West China Hospital Clinical Trials and Biomedical Ethics Committee, Sichuan University (Approval in 2023, No. 1064).

## Consent

Written informed consent was exempted given the retrospective nature of the study.

## Conflicts of Interest

The authors declare no conflicts of interest.

## Data Availability

The data that support the findings of this study are available from the corresponding author upon reasonable request.
